# Dr. Elizabeth Mabel Henderson

**Published:** 1911-05

**Authors:** 


Dr. Elizabeth Mabel Henderson, a graduate of Queen's University, Kingston, Ont., in 1892, died at her home in Hamilton, Ont., March 28, 1911, of heart disease. She was the gold medalist of her year at Queen's University.

